# Supplementary material for: What Solutions Exist for Developmental Delays Facing Indigenous Children Globally? A Co-Designed Systematic Review
Source: Children (Basel). 2020 Dec 10;7(12):285. doi: 10.3390/children7120285 (PMC7763469; doi:10.3390/children7120285)
Supplement: Supplementary file 1 [file children-07-00285-s001.pdf]

Supplementary

Table S1. Search strategy.

Search	Query
#1	Oceanic ancestry group OR Indigenous groups OR Aboriginal OR First Nation people OR Sami OR Indian OR American Native OR African ancestry group OR European ancestry group
#2	Preschool-aged children
#3	Neurological delay OR Developmental delay OR socio-emotional regulation OR stress disorder OR social competence OR hyperarousal OR language delay OR emotional trauma OR Posttraumatic stress disorder
#4	Therapeutics OR early intervention OR solution OR service OR program
#5	#1 AND #2 AND #3 AND #4
	.mp. [mp=title, abstract, heading word, table of contents, key concepts, original title, tests & measures]

Table S2: Critical Appraisal Skills Program (CASP) Assessment

**CASP Appraisal**	**Articles**						
Yes, Unsure, No	**Big Foot**	**De Rios**	**Fazel**	**Kellam**	**Morsette**	**Spencer**	**Vazir**
1. Clear Aims Statement	N	N	Y	Y	Y	Y	Y
2. Appropriate Qualitative methodology	N	N	N, Quantitative, but appropriate	N, Quantitative, but appropriate	N, Quantitative	N, Quantitative, but appropriate	N, Quantitative
3. Research design appropriate	Y	Y	Y	Y	N	Y	Y
4. Recruitment strategy	N/A	N/A	Y	Y	Y	Y	Y
5. Data collection appropriate	N/A	N/A	Y	Y	Y	Y	Y
6. Researchers and participants distinguished	N/A	N/A	N	Y	Cant tell	N	Y
7. Ethical issues	N/A	N/A	N	N	Cant tell	N	N
8. Rigorous Data analysis	N/A	N/A	Y	Y	Y	Y	Y
9. Clear statement of findings	N/A	N/A	Y	Y	Y	Y	Y
10. How valuable is the research	Y	Y	Y	Y	Y	Y	Y

N: No, Y: Yes, N/A: Not/Applicable
